# Expanding the Genetic Spectrum of Non-Syndromic Cleft Lip and Palate Through Whole-Exome Sequencing

**DOI:** 10.3390/ijms262412111

**Published:** 2025-12-16

**Authors:** Barbara Biedziak, Justyna Dąbrowska, Agnieszka Bogdanowicz, Karolina Karbowska, Adrianna Mostowska

**Affiliations:** 1 Department of Orthodontics and Craniofacial Anomalies, Poznan University of Medical Sciences, 60-812 Poznan, Poland; biedziak@ump.edu.pl (B.B.); abogdanowicz@ump.edu.pl (A.B.); karo.karbowska@gmail.com (K.K.); 2 Department of Biochemistry and Molecular Biology, Poznan University of Medical Sciences, 60-781 Poznan, Poland; jdabrowska@ump.edu.pl

**Keywords:** CL/P, etiology, WES, risk factors

## Abstract

Non-syndromic cleft lip with or without cleft palate (ns-CL/P) is one of the most common craniofacial anomalies with a multifactorial etiology. To investigate the contribution of rare variants to disease risk, we performed whole-exome sequencing (WES) in 58 patients with ns-CL/P from a homogeneous Polish population, excluding from analysis 423 previously investigated cleft candidate genes. After stringent filtering, prioritization, and segregation analysis, we identified 31 likely pathogenic (LP) variants across 30 genes, significantly enriched in categories related to developmental processes. Notably, 29% of variants occurred in genes not previously linked to clefting, including *AGO1*, *ARID1A*, *ATP1A1*, *FOXA2*, *GDF7*, *HOXB3*, *LRP5*, *MAML1*, and *ZNF319*. Three were de novo: FOXA2_p.Arg260Pro, MAML1_p.Gln65Ter, and ZNF319_p.Gln64Ter. Most of the remaining variants were inherited from unaffected parents, suggesting incomplete penetrance and possible modifier effects consistent with the heterogeneous etiology of ns-CL/P. Additionally, analysis of common variants in the 30 loci harboring rare LP variants revealed nominal associations with ns-CL/P for *NXN*, *EXT1*, *MAML1*, and *TP53BP2* loci. These results support the candidacy of these genes and suggest contributions from both rare and common variants. In conclusion, we report novel LP variants expanding the spectrum of candidate genes and providing new insights into the genetic landscape of orofacial clefts.

## 1. Introduction

Non-syndromic cleft lip with or without cleft palate (ns-CL/P) is the most frequent type of orofacial clefting, affecting approximately 1 in 1000 live births in European populations [1,2]. The condition is clinically heterogeneous and is usually subdivided into cleft lip (ns-CL) and cleft lip with cleft palate (ns-CLP). Evidence from embryology and epidemiology indicates that these subtypes are not identical in origin and may involve distinct genetic mechanisms [2,3]. Beyond the structural defect, ns-CL/P is accompanied by a wide range of medical and psychosocial problems. Affected individuals may experience feeding difficulties, recurrent ear infections, impaired speech, and malocclusion, while concerns about facial appearance and treatment burden add further challenges. Families, in turn, often face substantial psychological stress and financial strain [1,4]. Lifelong multidisciplinary care is typically required, beginning with surgical repair and extending through orthodontic, speech, and psychological interventions. For these reasons, ns-CL/P is not only a congenital malformation but also a public health concern with lasting consequences for quality of life and healthcare systems.

The etiology of ns-CL/P is widely recognized as multifactorial, involving the interplay of genetic and environmental factors [5,6]. In recent years, attention has also turned to epigenetic influences and non-coding RNAs. DNA methylation and histone modifications can alter the expression of key developmental genes, thereby influencing specific cleft subtypes [7,8,9,10]. Genes under such epigenetic control include those encoding transcription factors and signaling molecules [11]. MicroRNAs, such as miR-140, miR-149, and miR-300, have been implicated in fine-tuning gene expression programs required for craniofacial morphogenesis, and variants in miRNA genes have also been linked to ns-CL/P [12,13]. Together, these findings emphasize that epigenetic regulation is an important modifier of both genetic susceptibility and the observed phenotype.

Family and twin studies provide strong evidence for the heritability of ns-CL/P. Estimates suggest that 50–80% of liability is genetically determined [5,14]. First-degree relatives of affected individuals carry a substantially higher recurrence risk; for example, an up to 32-fold increase has been reported compared with the general population [15]. Concordance rates of 40–60% in monozygotic twins, compared with only 3–5% in dizygotic twins, further support a strong genetic influence, although not the sole determinant [16]. These data indicate an important hereditary component, but also suggest that incomplete penetrance and variable expressivity are shaped by genetic modifiers and environmental exposures.

Over the past two decades, numerous loci and candidate genes have been proposed as contributors to ns-CL/P based on linkage studies, candidate-gene analyses, and genome-wide association studies (GWAS) [2,6]. However, results are often inconsistent between populations, reflecting the disorder’s heterogeneity. The most reproducible findings across diverse populations involve *IRF6* (OMIM *607199) and the non-coding region of chromosome 8q24, both consistently confirmed as major risk loci [17,18,19]. Beyond these well-established signals, many additional genes and pathways have been implicated, but their effects often appear population-specific, underscoring the complexity of unraveling the genetic architecture of this malformation.

More recently, next-generation sequencing (NGS) has substantially expanded our understanding of the genetic basis of ns-CL/P, particularly by uncovering the contribution of rare variants with potentially large effects. Whole-genome sequencing (WGS), whole-exome sequencing (WES), and targeted multigene panels have revealed numerous rare and ultra-rare pathogenic or likely pathogenic (P/LP) variants in both established candidate genes and previously unsuspected loci [20,21,22,23,24]. These findings highlight the marked heterogeneity of ns-CL/P, with reported variants including de novo variants (DNVs), alleles inherited from affected parents, and variants transmitted by unaffected carriers. Rare variants with moderate or high penetrance are thought to act either as significant contributors and major drivers of the phenotype [25,26] or as genetic modifiers that may interact with environmental influences, contributing to phenotypic variability and expressivity. While common variants explain part of the heritability, rare coding and splicing variants constitute an equally important, though incompletely defined, source of genetic risk. Recent sequencing efforts also point to DNVs as a reservoir of novel candidate genes, adding another layer of complexity [27].

Despite substantial progress, the full genetic background of ns-CL/P remains unresolved. Many of the rare P/LP variants described so far are private to single patients or families, and numerous candidate genes have been reported only once without independent replication. Combined with strong population-specific effects, this heterogeneity hampers the establishment of a definitive catalog of ns-CL/P genes. To help address this gap, we previously analyzed 423 cleft genes in a Polish cohort using WES and detected rare coding and splicing variants in both established and novel loci, such as *EFTUD2* (OMIM *603892), *JAG1* (OMIM *601920), and *SHH* (*OMIM 600725), among others [21]. In the present study, we extend this analysis to all remaining coding genes captured by WES, with the aim of identifying additional rare variants that may influence susceptibility to ns-CL/P.

## 2. Results

### 2.1. Whole-Exome Sequencing (WES) Results

After initial variant selection and prioritization, 263 heterozygous single nucleotide variants (SNVs; 3–8 per patient) were designated for confirmation and segregation analyses. Based on the obtained results and annotation data for each variant and its gene, a final set of 31 LP variants in 30 genes was established. Detailed characteristics and in silico pathogenicity scores of all identified variants are provided in Table 1 and Table 2, while Tables S1 and S2 summarize the characteristics of the affected genes.

One of the identified variants, *VWA8* p.Arg668Gln (rs138075452), had previously been classified as LP for non-syndromic cleft lip and palate in the ClinVar database and was also reported in a previous study [28]. This variant, with a Combined Annotation Dependent Depletion (CADD) score of 27.9, was detected in a patient with ns-CLP and his affected father. Two other variants transmitted from affected parents were novel and located in *GDF7* (p.Leu361Phe; CADD = 27) and *FAT1* (p.Ile895AspfsTer2; MutPred-LOF = 0.54). Variants in these genes have not previously been associated with orofacial clefts in either humans or mice. A novel splicing variant in *LRP5* (c.3638-1G>A; adaptive boosting: ADA score 0.999) was detected in both siblings with ns-CLP but was inherited from their healthy mother.

Among the LP variants, three were confirmed de novo mutations with verified maternity and paternity. All three occurred in patients with ns-CLP. These included a missense variant in *FOXA2* (p.Arg260Pro, CADD = 35), predicted as pathogenic or deleterious by all applied tools, and two nonsense variants: *MAML1* (p.Gln65Ter; MutPred-LOF = 0.50) and *ZNF319* (p.Gln64Ter; MutPred-LOF = 0.45). All three genes were classified as likely or very likely dominant and had not been previously implicated in clefting.

The remaining LP variants consisted of novel or ultra-rare missense variants (minor allele frequency: MAF ranging from 0.00 to 2.20 × 10^−5^) and one frameshift variant, all inherited from healthy parents. The missense variants with the highest CADD scores (≥32) included *ARID1A* p.Arg2143His (rs2124148967), *CTNNA1* p.Ser810Phe, and *MYH3* p.Arg18Trp (rs750940457). In total, nine missense variants were identified in genes previously implicated in ns-CL/P: *CTNNA1*, *EFNB2*, *HIF1A*, *LAMA5*, *IFT172*, *MYH3*, *RYR1* (two variants), and *TENM4* [22,29,30,31,32,33,34,35]. The two *RYR1* variants (p.Leu3046Val, rs2145659189; p.Asn3066Lys, rs201863144) occurred in cis in the same patient. Five genes harboring missense variants (*ATN1*, *IFT172*, *MYH3*, *NXN*, and *RYR1*) had previously been associated with syndromic forms of clefts, and 13 (*EFNB2*, *EXT1*, *HAND1*, *IFT172*, *KRT17*, *MEN1*, *NCOR2*, *NXN*, *ROBO1*, *RPGRIP1L*, *RYR1*, *SLC32A1*, and *TP53BP2*) were reported to cause orofacial clefts in knockout mouse models.

Among the 30 genes identified in the present study, the only genes previously associated with both syndromic and non-syndromic clefts in humans and mice were *RYR1* and *IFT172*. In the latter, a missense variant, p.Asp1366Tyr (rs776240963), was detected in a patient with CLP and a bifid left renal pelvis. This SNV was predicted to be pathogenic by most in silico tools (CADD = 26.3).

Among the genes harboring LP variants, 22 were predicted to follow a dominant inheritance pattern (classified as likely or very likely dominant by the machine learning tool DOMINO) [38]. Of the remaining genes, seven were predicted to follow an autosomal recessive mode of inheritance, while three could be consistent with either dominant or recessive inheritance (Table 2).

Analysis of protein structures using the UniProt knowledgebase (https://www.uniprot.org/, accessed on 1 July 2025) revealed that 11 out of 26 missense variants were located within functional domains or domain superfamilies of the corresponding proteins (Table S3).

### 2.2. Results of Common Variants Analysis—GWAS Data

Analysis of 2151 common SNVs located within the 30 gene loci identified in the present study revealed four variants significantly associated with the risk of ns-CL/P (Table 3, Figures S1–S4). The strongest signal was observed for an intronic variant in the *NXN* gene (rs8081951), with a *p*_trend-_value of 2.52 × 10^−4^ (odds ratio: OR = 1.62). Three additional variants were: an intergenic SNV (rs28564876) located ~81.8 kb upstream of *MAML1* (p_trend_ = 9.34 × 10^−4^), synonymous variant in *EXT1* (*p*_trend_ = 9.56 × 10^−4^), and an intergenic SNV upstream of *TP53BP2* (*p*_trend_ = 1.16 × 10^−3^). The allelic ORs for these variants ranged from 0.70 to 1.80. All four variants remained statistically significant after Bonferroni correction for the number of tested genes (0.05/30), but none reached the stricter threshold accounting for the total number of tested SNVs (*p*_trend_ < 2.32 × 10^−5^). Analysis of the remaining SNVs within these gene loci did not show any additional variants significantly associated with ns-CL/P risk. However, the linkage disequilibrium (LD) block encompassing the *TP53BP2* gene is relatively broad, consistent with a region of low recombination, and contains several variants that approach statistical significance. This LD structure suggests that the observed signal may reflect a true association driven by multiple correlated SNVs within the locus.

### 2.3. Enrichment of Gene Ontology (GO) Categories

Over representation analysis (ORA) identified 24 GO biological process categories significantly enriched among the tested genes (*p*_adj_ < 0.05). The top five enriched terms were “tissue morphogenesis”, “embryonic morphogenesis”, “animal organ morphogenesis”, “morphogenesis of an epithelium”, and “circulatory system development” (all *p*_adj-_values ≤ 8.52 × 10^−6^; Table 4). No GO molecular function categories were enriched among analyzed cleft genes (Table S4). Although the majority of genes were annotated with broad molecular function terms such as “protein binding”, these categories were not statistically enriched in ORA due to their high prevalence in the genome-wide background. A graphical overview of GO Slim terms and the hierarchical relationships of significantly enriched biological process categories is provided in Figures S5 and S6.

## 3. Discussion

It is well established that genetic factors, including both common and rare nucleotide variants, play a significant role in the complex etiology of ns-CL/P. In this study, we applied WES combined with GWAS data to investigate genetic determinants of this common craniofacial anomaly in a cohort from the genetically homogenous Polish population. Through a stringent, multistage filtering strategy, we identified 31 novel and ultra-rare LP coding and splicing variants in 30 genes that may potentially be involved in the pathogenesis of ns-CL/P. While the contribution of these variants remains to be functionally validated, our findings suggest the involvement of both previously implicated genes and novel candidates, supporting the heterogeneity and polygenic nature of this condition.

One of the most noteworthy observations from our study was the detection of three DNVs in patients with ns-CLP: a highly deleterious missense variant in *FOXA2* and two nonsense mutations in *MAML1* and *ZNF319*. All three genes are predicted to act in an autosomal dominant manner and have not previously been associated with either syndromic or non-syndromic forms of clefting. *FOXA2* encodes a transcription factor essential for early embryogenesis and endodermal organogenesis [40,41]; its interaction with the Sonic Hedgehog (Shh) pathway provides a plausible link to craniofacial development [42,43,44]. While no defined human syndrome has yet been attributed to *FOXA2* mutations in OMIM, variants in this gene have been associated with hypopituitarism, hyperinsulinism, craniofacial abnormalities, and midline defects, including a reported case of paramedian cleft lip and palate in a patient carrying a ~5.4 Mb deletion at 20p11.2 encompassing 35 genes, among them *FOXA2* [45,46]. MAML1 functions as a transcriptional coactivator in Notch and other pathways, including p53, β-catenin, and Shh signaling, all of which are essential for craniofacial morphogenesis and palatogenesis [47,48,49,50,51]. In addition, Maml1 has been shown to enhance Runx2-dependent osteoblast and chondrocyte differentiation in mice, a process relevant to secondary palatal ossification rather than primary lip fusion [52]. Thus, while the precise mechanism by which MAML1 variants contribute to cleft lip and palate remains uncertain, its involvement in multiple signaling pathways critical for craniofacial development makes it a plausible candidate gene. ZNF319, although less well characterized, has been implicated in transcriptional regulation and Wnt signaling, another pathway central to craniofacial morphogenesis [43,44]. This zinc finger transcription factor has been proposed to act as a tumor suppressor regulating cell cycle progression and proliferation [53].

DNVs are an important category of genetic variation that may contribute to multifactorial disorders, including ns-CL/P [54]. Since these variants are not inherited and therefore not subject to purifying selection, they have a greater probability of exerting strong functional effects [55]. Evidence supporting a contribution of DNVs to ns-CL/P comes from trio-based sequencing studies. In the first large-scale WGS study of orofacial clefts, Bishop et al. reported a significant enrichment of loss-of-function DNVs in genes highly expressed in craniofacial tissues across multi-ethnic case–parent trios [26]. More recently, Ishorst et al. showed that integrating DNV detection with polygenic risk score analysis is a powerful approach to gene discovery in ns-CL/P, highlighting recurrent DNVs in *MDN1* and *PAXIP1* [27]. In line with these findings, previous studies in the Polish population have identified LP DNVs in *CTNND1*, *NOTCH2*, *FGFR1*, and *KMT2D* in patients with non-syndromic clefts [21]. Together, these observations suggest that DNVs represent one of several mechanisms contributing to ns-CL/P susceptibility. In our cohort, three LP DNVs (*FOXA2*, *MAML1*, *ZNF319*) emerged as preliminary candidate variants; however, each was observed only once and is classified as likely pathogenic or variants of uncertain significance under ACMG criteria [56] (Table 2). Robust gene–disease associations require recurrent independent findings across multiple studies. Therefore, the DNVs identified here should be considered exploratory, and validation in larger cohorts with functional follow-up will be essential to clarify their pathogenic relevance.

Another noteworthy finding was a missense variant in the *VWA8* gene. The p.Arg668Gln substitution (rs138075452) has previously been reported as likely pathogenic for ns-CL/P and is cataloged in ClinVar [28]. Aylward et al. identified five candidate variants in VWA8, each present in a single family, but none showed complete segregation with ns-CL/P [28]. The recurrence of the *VWA8* p.Arg668Gln variant in our cohort, together with its inheritance from an affected father, supports its potential role as a risk factor. In addition, we identified two other LP variants transmitted from affected parents, including *GDF7* p.Leu361Phe and *FAT1* p.Ile895AspfsTer2. Although these SNVs, classified as variants of uncertain significance, occur in genes not previously linked to orofacial clefts, their inheritance from affected parents is of particular interest. Additional lines of evidence, such as epigenetic and expression data, provide supportive but not conclusive indications of a possible role of these candidate genes in craniofacial development. Importantly, differential methylation of *FAT1* has been implicated in ns-CL/P [7], pointing to epigenetic dysregulation of this gene as one of the potential disease mechanisms. These results align with emerging evidence that epigenetic regulation of craniofacial genes, including altered methylation patterns, may contribute to ns-CL/P risk. *FAT1* encodes an atypical cadherin that participates in the planar cell polarity pathway, known to play a role in craniofacial morphogenesis [57]. Moreover, *FAT1* can activate multiple signaling cascades, such as Wnt/β-catenin, Hippo, and MAPK/ERK, which regulate essential cellular processes such as proliferation, migration, and invasion [58]. Support for *GDF7* involvement comes from mouse studies, where transcriptomic analyses of upper lip and primary palate development revealed *Gdf7* expression in craniofacial structures [59].

The remaining novel and ultra-rare LP variants identified in this study were inherited from healthy parents. Nine of them occurred in genes previously associated, or suspected to be associated with ns-CL/P risk based on human genetic studies (*CTNNA1*, *EFNB2*, *HIF1A*, *LAMA5*, *IFT172*, *MYH3*, *RYR1* and *TENM4*) [22,29,30,31,32,33,34,37], further supporting their candidacy for this anomaly. Notably, *MYH3* has also been implicated in syndromic forms of clefting (OMIM #178110, #618469), while both *RYR1* and *IFT172* have been linked to syndromic and non-syndromic clefts in humans and mice. Among these, *IFT172* appears particularly noteworthy, as we identified a missense variant, p.Asp1366Tyr (rs776240963), located in the tetratricopeptide-like helical domain of the protein, in a patient with CLP and a bifid left renal pelvis but no other congenital malformations. *IFT172* encodes a key component of the intraflagellar transport complex, which is critical for cilia formation and maintenance [60]. Defects in this process underlie a group of disorders known as ciliopathies, many of which present with overlapping craniofacial, renal, and skeletal anomalies [61,62]. Indeed, pathogenic *IFT172* variants have been reported in syndromic conditions such as Mainzer–Saldino and Bardet–Biedl syndromes, which can include clefting and renal malformations as part of the clinical spectrum [62]. Mouse knockout studies further confirm its role, as *Ift172*-deficient embryos display a broad spectrum of developmental abnormalities, including craniofacial defects (abnormal craniofacial morphology and cleft secondary palate) and kidney malformations. Overall, our findings provide additional evidence that disruption of ciliary function, through genes such as *IFT172*, may represent a shared developmental mechanism underlying both craniofacial and renal anomalies.

Among other variants inherited from unaffected parents were those in genes previously associated with syndromic forms of clefting in humans (*ATN1*), genes whose involvement in clefting has been demonstrated in knockout mouse models (*EXT1*, *HAND1*, *KRT17*, *MEN1*, *NCOR2*, *ROBO1*, *SLC32A1*, and *TP53BP2*), as well as genes included in both categories (*NXN* and *RPGRIP1L*). In this group, variants were also detected in genes for which, to our knowledge, no direct association with orofacial clefts has been reported so far. These include *AGO1*, *ARID1A*, *ATP1A1*, *HOXB3* and *LRP5*. Notably, in *AGO1*, which encodes an Argonaute protein essential for miRNA processing and RNA-mediated gene silencing [63], an amino acid substitution p.Ser608Phe (rs1353927503) was identified within the functional PIWI domain. Recently, pathogenic variants and gross deletions involving *AGO1* have been associated with neurodevelopmental disorders in humans, including autism spectrum disorder, developmental delay, intellectual disability, and dysmorphic facial features [64]. Some affected individuals also presented with high-arched palate and dental crowding [65]. Given that microRNAs play a critical role in regulating gene expression during craniofacial development, and that SNVs in miRNA genes have been implicated in ns-CL/P risk, *AGO1* emerges as a strong novel candidate for clefting. Other noteworthy findings include *LRP5*, which encodes a component of the Wnt-Fzd-LRP5-LRP6 complex that activates β-catenin signaling [66], and *HOXB3*, a sequence-specific transcription factor that provides positional identity along the anterior–posterior axis during development [67]. In a regulatory network analysis of cleft genes, Li et al. reported *HOXB3* enrichment in pathways related to positive regulation of cell division [68]. Notably during upper lip formation the lateral nasal process undergoes a peak of cell proliferation, and perturbations in growth at this critical stage may impair fusion [69]. Experimental studies have also demonstrated that Hoxb3 directly regulates Jag1 expression in the pharyngeal epithelium, thereby affecting neural crest cell migration and pharyngeal arch morphogenesis, further supporting a possible role in craniofacial development [70]. While these observations suggest a potential indirect involvement of HOX3B, direct evidence of its role in craniofacial regions relevant to lip development is lacking, and its contribution to CLP pathogenesis therefore remains speculative.

Inheritance of LP variants from clinically unaffected parents, as observed for the majority of variants in this study, suggests incomplete penetrance and variable expressivity. Such variants may also act as genetic modifiers that influence individual risk, supporting the multifactorial model of ns-CL/P etiology in which multiple genetic variants, in combination with epigenetic and environmental exposures, collectively determine the likelihood of disease manifestation [71]. Environmental factors implicated in ns-CL/P include both unmodifiable factors (e.g., ancestry, sex, family history) and modifiable maternal exposures during the periconceptional period [72]. Maternal health and lifestyle factors, such as tobacco smoking, alcohol consumption, nutritional deficiencies, obesity, febrile maternal illness, and certain medications, may alter the intrauterine environment and potentiate the phenotypic effects of inherited variants [73,74,75,76,77,78,79]. Environmental toxicants, including disinfection byproducts or nitrates in drinking water [80], have also been linked to an increased risk in epidemiological studies. Within this framework, LP variants may not be restricted to affected individuals. They can also be present in unaffected family members, highlighting the complex gene-gene and gene-environment interactions underlying cleft pathogenesis. Unfortunately, our study did not account for environmental exposures.

To further contextualize our findings, we analyzed common variants within the 30 gene loci harboring rare LP variants. At the gene level, four SNVs showed nominal associations with ns-CL/P, including intronic and synonymous variants in *NXN* and *EXT1*, as well as intergenic variants in the *MAML1* and *TP53BP2* loci. These results should be interpreted with caution, as none of these associations remained statistically significant after correction for multiple testing across the total number of variants analyzed. Nevertheless, they support the candidacy of these four genes and suggest that both common and rare variants within them may contribute to ns-CL/P risk. The strongest signal was observed for an intronic variant in *NXN*, which encodes a redox-dependent negative regulator of the Wnt signaling pathway [81]. Notably, the detection of a rare LP in the thioredoxin domain of the NXN protein (p.Lys168Thr) in our cohort provides converging lines of evidence suggesting a potential role of this gene in ns-CL/P pathogenesis. Validation of these associations and clarification of their biological relevance will require larger, well-powered studies conducted across diverse populations.

Finally, we performed over-representation analysis of GO categories to better understand the biological significance of the identified genes. This analysis revealed several biological process terms that were significantly enriched, with the strongest signals observed for categories broadly related to developmental processes. The top enriched categories included tissue morphogenesis, embryonic morphogenesis, animal organ morphogenesis, morphogenesis of an epithelium, and circulatory system development. These findings are consistent with previous studies that have linked cleft-associated genes to key developmental processes and signaling pathways [23,30]. Nevertheless, it should be noted that our analysis excluded the 423 cleft candidate genes examined in our previous study [21]. Thus, the enrichment patterns presented here should be interpreted as complementary to, rather than directly comparable with, those reported for cleft genes in earlier analyses.

This study has several limitations that should be acknowledged. First, although WES is effective for detecting coding variants, it does not capture non-coding regions, structural rearrangements, or copy number variants that are known to contribute to orofacial clefting [82,83]. Second, the identified variants were assessed only through in silico prediction tools, and no functional assays were carried out to confirm their pathogenicity. Third, our findings were not replicated in an independent cohort from a different population, which limits their generalizability. Although the genetic homogeneity of the Polish population may have facilitated rare variant discovery, replication in larger and ethnically diverse cohorts or through cross-population meta-analyses will be required to confirm the broader relevance of the identified variants. Fourth, parental data were restricted to targeted resequencing of selected variants for segregation analysis, rather than trio-based WES, which precluded comprehensive detection of DNVs and Mendelian errors—an approach recognized to be effective for identifying indels [83]. Fifth, our filtering and prioritization strategy may not fully reflect the true biological impact of variants and could have resulted in the omission of clinically relevant changes. Sixth, the statistical power of the GWAS-based analysis was limited, as the applied multiple testing correction considered only the number of tested genes rather than the total number of SNVs, thereby increasing the risk of false-positive findings. Finally, the overall sample size of our study was relatively small, which reduced the power to detect rare variants.

In addition, the present study analyzed ns-CL and ns-CLP as a combined group, in line with previous genetic investigations, to maximize power given the limited sample size. We acknowledge, however, that cleft lip only and cleft lip with palate are clinically and embryologically distinct subtypes that may involve partially different genetic mechanisms [2,3]. Epidemiological studies indicate that ns-CL and ns-CLP differ in sex distribution, laterality, and recurrence risk, while embryological evidence suggests that lip and palate closure may involve distinct morphogenetic processes and signaling pathways [5,43,84]. Notably, most rare variants identified in our cohort occurred in ns-CLP patients, with only four variants detected in ns-CL cases. Larger studies will be required to clarify whether the genetic risk factors observed here are specific to ns-CLP or also extend to ns-CL.

## 4. Materials and Methods

### 4.1. Workflow of the Study

**Figure 1:**
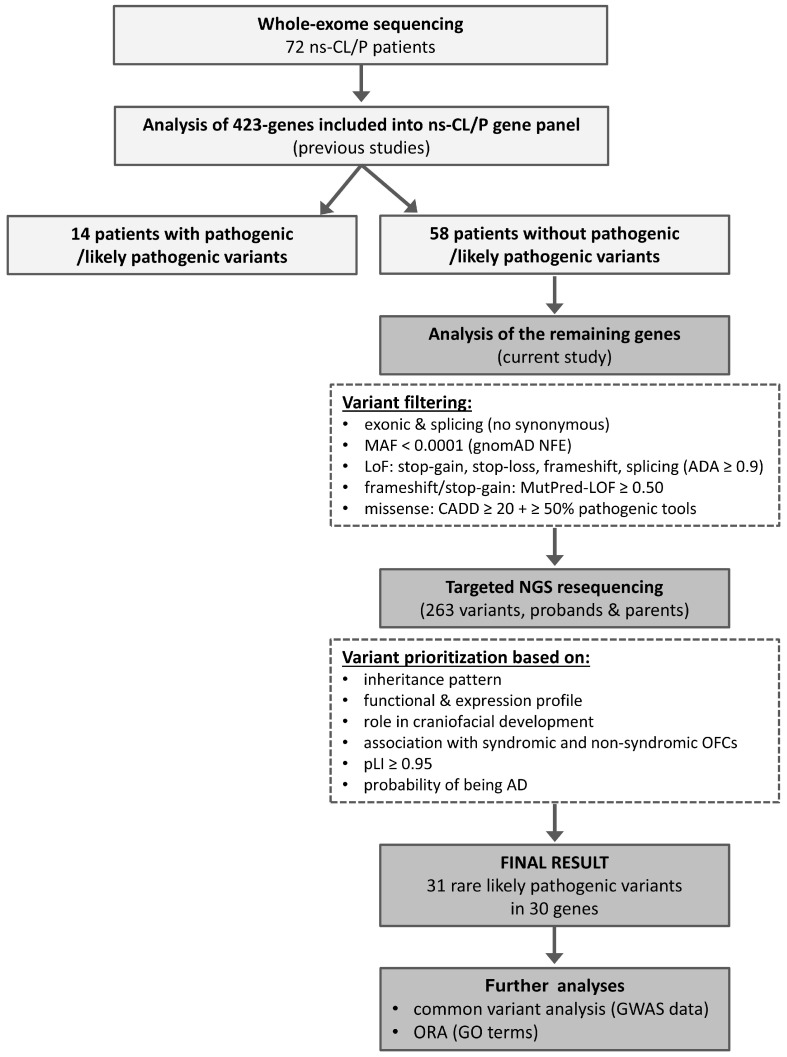
Workflow of variant detection and prioritization in the current study.

WES was performed for 72 patients with ns-CL/P. Parental DNA samples were available for all patients and were used for targeted NGS resequencing of selected variants to assess segregation, but not for trio-based WES. In the first step, variants were analyzed exclusively in 423 genes included in a previously established hypothesis-driven ns-CL/P multigene panel. Importantly, in our earlier publication, this panel was interrogated in two ways: the selected 423 genes’ coding exons and exon–intron boundaries were screened using either an NGS-based multigene panel (in 56 patients) or WES (in 72 patients), although only variants within this targeted gene set were analyzed and reported [21]. Briefly, among the 72 patients tested by WES, rare P/LP variants were identified in 14 individuals, whereas the remaining 58 carried no such variants. In the present study, we focused on these 58 patients and extended the analysis to all other coding genes captured by WES. After variant filtering and prioritization, 263 SNVs were confirmed and tested for segregation in probands and their parents by targeted NGS resequencing. Further prioritization yielded a final dataset of 31 LP variants in 30 genes. In the next stage, common variants in genes carrying rare LP variants were examined using data from our previously published GWAS of ns-CL/P [39,85]. This GWAS was performed in a completely independent Polish cohort comprising 269 ns-CL/P patients and 569 controls, genotyped using the HumanOmniExpressExome-8 v1 array (Illumina, San Diego, CA, USA). Finally, ORA was conducted for Gene Ontology categories (Figure 1).

The detailed selection and characterization of the 423 genes included in the ns-CL/P multigene panel have been described previously [21,86]. In brief, the panel encompassed: established ns-CL/P candidate genes, genes associated with syndromic clefts, genes whose knockout in mice results in orofacial clefts, genes encoding proteins involved in signaling pathways critical for craniofacial development, and genes with high expression in embryonic craniofacial tissues (Table S5).

Whole-exome sequencing (WES) was performed for 72 patients with non-syndromic cleft lip with or without cleft palate (ns-CL/P). Variants in 423 genes included in a previously published ns-CL/P multigene panel were analyzed first, identifying pathogenic or likely pathogenic (P/LP) variants in 14 patients. The remaining 58 patients underwent extended analysis of all other coding genes captured by WES. After variant filtering, 263 single-nucleotide variants were confirmed and tested for segregation in probands and their parents using targeted resequencing. Subsequent prioritization yielded 31 LP variants in 30 genes. In the next stage, common variants in genes carrying rare LP variants were examined using data from our previously published Genome Wide Association Study (GWAS) of ns-CL/P, followed by overrepresentation enrichment analysis (ORA). Abbreviations: AD, autosomal dominant; CADD, Combined Annotation Dependent Depletion; GO, gene ontology; LoF, loss of function; MAF, minor allele frequency; NFE, European non-Finnish; pLI, probability of being loss-of-function intolerant.

### 4.2. Study Population

The study cohort comprised 56 unrelated patients and one sibling pair (total n = 58) diagnosed with ns-CL/P, aged 1–15 years. All participants were enrolled in the Comprehensive Therapy Programme for Craniofacial Anomalies at Poznan University of Medical Sciences. The cohort consisted of 48 individuals with ns-CLP (82.8%), and 10 individuals had ns-CL (17.2%). The overall male-to-female ratio was 1.64:1 (Table 5). Minor associated anomalies were observed in two patients (3.5%), specifically kidney malformations such as a bifid left renal pelvis or horseshoe kidney. These patients were retained in the cohort, as no known syndromes were diagnosed after a detailed review of their medical records. A positive family history of ns-CL/P was reported in 19 cases (32.8%).

Peripheral blood samples were collected from patients and their parents. All participants were Caucasians of Polish origin. Genomic DNA was extracted from peripheral blood lymphocytes using the salting-out method. The study was approved by the Institutional Review Board of Poznan University of Medical Sciences, Poland (approval number 1115/18, approval date: 7 November 2018). Written informed consent was obtained from all adult participants and from the parents or legal guardians of underage patients, in accordance with the Declaration of Helsinki [87].

### 4.3. Whole Exome Sequencing (WES)

Library preparation for WES was performed using the TruSeq DNA Exome kit (Illumina Inc., San Diego, CA, USA). Sequencing was conducted on an Illumina HiSeq 4000 platform with paired-end reads (2 × 100 bp). Raw data were processed following a previously described pipeline [21,84]. The mean on-target sequencing depth was 95.6x, with an average of 97.6% (range: 94.6–99.2%) of target bases covered at ≥20x. Raw sequencing data were demultiplexed and converted to FASTQ format using bcl2fastq (Illumina Inc., San Diego, CA, USA). After adapter trimming and removal of low-quality reads, sequences were aligned to the GRCh38/hg38 reference genome using the Burrows–Wheeler Aligner (http://bio-bwa.sourceforge.net/, accessed on 1 February 2017). The Genome Analysis Toolkit (GATK, https://www.broadinstitute.org/gatk/, accessed on 1 February 2017) was used for calling SNVs and short insertions/deletions (indels). Variants were annotated with functional information, in silico pathogenicity predictions, population allele frequency (gnomAD European non-Finnish population, http://gnomad.broadinstitute.org/, accessed on 1 June 2025), and clinical significance based on ClinVar (https://www.ncbi.nlm.nih.gov/clinvar/, accessed on 1 June 2025), the Human Gene Mutation Database (HGMD, http://www.hgmd.cf.ac.uk, accessed on 1 June 2025), and Online Mendelian Inheritance in Man (OMIM, https://omim.org/, accessed on 1 June 2025). Phenotypic information from knockout mouse models was retrieved from the Mouse Genome Informatics database (https://www.informatics.jax.org/, accessed on 1 June 2025).

Predicted variant effects were evaluated using 22 in silico tools. These included individual predictors: SIFT, SIFT4G, PolyPhen-2_HDIV, PolyPhen-2_HVAR, MutationTaster, MutationAssessor, PROVEAN, M-CAP, MutPred2, PrimateAI, DEOGEN2, ClinPred, LIST-S2, ESM1b, AlphaMissense, fathmm-XF, as well as meta-predictors: MetaSVM, MetaLR, MetaRNN, REVEL, BayesDel_addAF, BayesDel_noAF. All scores were retrieved from the dbNSFP database (https://www.dbnsfp.org/home, accessed on 1 July 2025). Additionally, CADD, Deleterious Annotation of genetic variants using Neural Networks (DANN), and ADA scores were used to assess missense and potentially splice-altering variants. The pathogenicity and functional impact of frameshift and stop-gain variants were predicted using the MutPred-LOF application (http://mutpred.mutdb.org/, accessed on 1 July 2025). Finally, gene-level metrics, including the probability of being loss-of-function intolerant (pLI) and the likelihood of autosomal dominant disease association, were obtained from gnomAD v4.1.0 and the DOMINO tool (https://domino.iob.ch/index.php, accessed on 1 July 2025).

### 4.4. Variant Selection and Prioritizing

Only exonic and splicing variants (excluding synonymous substitutions) with MAF < 0.0001 in the gnomAD European non-Finnish population were considered. Likely gene-disrupting variants included stop-gain, stop-loss, frameshift, splicing variants with an ADA score ≥ 0.9, as well as missense variants with a CADD score ≥ 20 that were classified as pathogenic by at least half of the applied in silico prediction tools. For frameshift and stop-gain variants, a MutPred-LOF score ≥ 0.50 was used as the threshold for likely pathogenicity.

After confirmation and segregation analysis, the final set of variants was selected based on inheritance patterns and the functional and expression profiles of the corresponding genes. Priority was given to variants identified in genes previously associated with non-syndromic or syndromic orofacial clefts, genes with a documented role in lip and palate development, and genes with pLI ≥ 0.95 and classified as likely and very likely dominant.

### 4.5. Confirmation and Segregation Analyses

Rare LP variants identified in the initial filtering step were validated and tested for co-segregation by targeted NGS resequencing in probands and their parents. Amplicon-based deep sequencing was carried out using the Nextera XT Kit (Illumina Inc., San Diego, CA, USA), followed by paired-end sequencing (2 × 100 bp) on an Illumina platform.

### 4.6. Common Variants Analysis—GWAS Data

Data on common SNVs within genes identified in the current study (n = 30) were extracted from our previously published GWAS of ns-CL/P in the Polish population [83,84]. Importantly, the GWAS was conducted in an independent ns-CL/P patient cohort, distinct from the WES cohort analyzed here; a detailed description of the patient and control groups is provided in the original publications. For each gene locus (gene ± 100 kb), associations of common SNVs with MAF ≥ 0.05 were tested with the Cochran-Armitage trend test. The strength of association was expressed as allelic OR. To correct for multiple testing, Bonferroni correction was applied, and *p*_trend_-values < 1.67 × 10^−3^ (0.05/30 genes) were considered statistically significant. Regional association plots for tested SNVs were generated using LocusZoom v1.1 tool (http://locuszoom.org/, accessed on 1 July 2025).

### 4.7. Over Representation Analysis (ORA)

For genes harboring rare LP variants, ORA of GO terms was performed using the WebGestalt tool (WEB-based Gene SeT AnaLysis Toolkit, http://www.webgestalt.org/, accessed on 1 June 2025), with all protein-coding genes in the genome as the reference set. GO categories, including biological process and molecular function, were considered significantly enriched at a Bonferroni-adjusted *p*_adj_-value < 0.05.

## 5. Conclusions

In conclusion, we report novel and rare LP variants that may contribute to ns-CL/P, including three DNVs in genes not previously associated with cleft risk. By integrating evidence from both rare and common variant analyses, our results support the multifactorial etiology of ns-CL/P, in which genetic and environmental factors may interact. These findings expand the list of candidate genes and suggest new directions that may explain the molecular basis of orofacial clefting. Future studies in larger patient cohorts from diverse populations and functional validation will be crucial to confirm our findings and to fully characterize the genetic landscape of ns-CL/P.

## Figures and Tables

**Table 1:** Rare likely pathogenic nucleotide variants identified in ns-CL/P patients.

Patient	Gender	Type of Cleft ^a^	Family History ^b^	Gene	Variant Effect			gnomAD	Genotype ^d^			Previous Gene Association with OFC ^e^		
					DNA	Protein	rs Number	Frequency ^c^	Patient	Father	Mother	Syndromic	Non-Syndromic	KO Mice
**DE NOVO VARIANTS**														
CLP_1	M	**CLP/R**	NO	***FOXA2***	c.779G>C	p.Arg260Pro	novel	NR	HET	wt	wt			
CLP_2	M	**CLP/R**	NO	***MAML1***	c.193C>T	p.Gln65Ter	novel	NR	HET	wt	wt			
CLP_3	M	**CLP/RL**	NO	***ZNF319***	c.190C>T	p.Gln64Ter	novel	NR	HET	wt	wt			
**VARIANTS INHERITED FROM AFFECTED PARENT**														
CLP_4	M	**CLP/RL**	YES—father	***GDF7***	c.1081C>T	p.Leu361Phe	novel	NR	HET	HET	wt			
CLP_5	M	**CLP/R**	YES—mother	***FAT1***	c.2681dupA	p.Ile895AspfsTer2	novel	NR	HET	wt	HET			
CLP_6	M	**CLP/RL**	YES—father	***VWA8***	c.2003G>A	p.Arg668Gln	rs138075452	4.72 × 10^−3^	HET	HET	wt		X [28]	
**VARIANTS INHERITED FROM HEALTHY PARENT**														
CLP_7	F	**CLP/RL**	YES—ex. paternal fam.	***CTNNA1***	c.2429C>T	p.Ser810Phe	novel	NR	HET	HET	wt		X [29,30]	
CLP_8	M	**CLP/L**	YES—ex. maternal fam.	***EFNB2***	c.590G>T	p.Ser197Ile	rs250151379	NR	HET	wt	HET		X [30]	X
CLP_9	F	**CLP/L**	YES—ex. maternal fam.	***HIF1A***	c.806G>A	p.Arg269Gln	rs2044522437	6.78 × 10^−6^	HET	wt	HET		X [31]	
CLP_10	M	**CLP/RL**	YES—ex. family	***LAMA5***	c.3520_3521delAG	p.Arg1174AlafsTer19	novel	NR	HET	HET	wt		X [22]	
CLP_11	M	**CLP/L**	YES—sister ^f^	***LRP5***	c.3638-1G>A		novel	NR	HET	wt	HET			
CLP_12	F	**CLP/L**	YES—brother ^f^											
CLP_13	F	**CL/R**	NO	***AGO1***	c.1823C>T	p.Ser608Phe	rs1353927503	8.48 × 10^−7^	HET	wt	HET			
CLP_14	M	**CLP/L**	NO	***ARID1A***	c.6428G>A	p.Arg2143His	rs2124148967	1.70 × 10^−6^	HET	wt	HET			
CLP_15	M	**CL/R**	NO	***ATN1***	c.2239C>A	p.Pro747Thr	rs782670138	7.85 × 10^−6^	HET	HET	wt	X		
CLP_16	M	**CLP/L**	NO	***ATP1A1***	c.1934C>T	p.Ala645Val	rss2101057973	NR	HET	HET	wt			
CLP_17	M	**CLP/RL**	NO	***EXT1***	c.493C>G	p.Gln165Glu	rs2130043213	NR	HET	wt	HET			X
CLP_18	M	**CLP/L**	NO	***HAND1***	c.328A>C	p.Ile110Leu	rs905545828	1.70 × 10^−6^	HET	HET	wt			X
CLP_19	F	**CLP/RL**	NO	***HOXB3***	c.374T>G	p.Leu125Arg	rs2068774883	NR	HET	wt	HET			
CLP_20 ^g^	F	**CLP/R**	NO	***IFT172***	c.4096G>T	p.Asp1366Tyr	rs776240963	5.09 × 10^−6^	HET	wt	HET	X	X [32]	X
CLP_21	M	**CL/R**	NO	***KRT17***	c.695A>G	p.Gln232Arg	rs1484519975	1.7 × 10^−6^	HET	wt	HET			X
CLP_22	M	**CLP/RL**	NO	***MEN1***	c.1565G>T	p.Arg522Leu	novel	NR	HET	wt	HET			X
CLP_23	F	**CL/R**	NO	***MYH3***	c.52C>T	p.Arg18Trp	rs750940457	5.93 × 10^−6^	HET	wt	HET	X	X [33,34]	
CLP_24	M	**CLP/RL**	NO	***NCOR2***	c.650C>T	p.Pro217Leu	rs199588853	2.72 × 10^−5^	HET	wt	HET			X
CLP_25	F	**CLP/L**	NO	***NXN***	c.503A>C	p.Lys168Thr	novel	NR	HET	wt	HET	X		X
CLP_26	F	**CLP/R**	NO	***ROBO1***	c.2026C>T	p.Arg676Trp	novel	NR	HET	HET	wt			X
CLP_27	F	**CLP/RL**	NO	***RPGRIP1L***	c.413A>G	p.Gln138Arg	rs2544706158	NR	HET	wt	HET	X		X
CLP_28	M	**CLP**	NO	***RYR1***	c.9136C>G ^h^	p.Leu3046Val	rs2145659189	0.00	HET	HET	wt	X [35,36]	X [33,37]	X
				***RYR1***	c.9198C>G ^h^	p.Asn3066Lys	rs201863144	0.00	HET	HET	wt			
CLP_29	F	**CLP/RL**	NO	***SLC32A1***	c.466G>T	p.Ala156Ser	rs2515237904	NR	HET	HET	wt			X
CLP_30	F	**CLP/R**	NO	***TENM4***	c.7916G>A	p.Arg2639Gln	rs775067963	2.20 × 10^−5^	HET	HET	wt		X	
CLP_31	F	**CLP/L**	NO	***TP53BP2***	c.1757C>A	p.Ala586Asp	rs2464444021	NR	HET	wt	HET			X

^a^ CL, cleft lip; CLP, cleft lip and palate; L, left side; R, right side. For one sample (CLP_28), the side of the cleft is unavailable. ^b^ ex., extended family member; fam., family. ^c^ The Genome Aggregation Database v4.1.0 (gnomAD; European non-Finnish population). NR, not reported. ^d^ Based on targeted next-generation sequencing. HET, heterozygous; wt, wild type. ^e^ OFC, orofacial clefts. *Syndromic*—gene previously associated with syndromic forms of OFC (https://www.omim.org/, accessed on 1 June 2025); *Non-syndromic*—gene previously associated with non-syndromic forms of OFC (https://pubmed.ncbi.nlm.nih.gov/, accessed on 1 June 2025); *KO Mouse*—cleft-related phenotype observed in knockout (KO) mice (https://www.informatics.jax.org/, accessed on 1 June 2025). Additional details in Tables S1 and S2. ^f^ Sibling cases. ^g^ Associated anomaly: bifid left renal pelvis. ^h^ Variants identified in cis. “X” indicates that a previous association for the given gene has been reported.

**Table 2:** Pathogenicity predictions of rare likely pathogenic nucleotide variants identified in ns-CL/P patients.

GENE				VARIANT		ACMG Classification ^c^	In silico Pathogenicity Prediction ^d^																						ADA	CADD	DANN	MutPred-LOF ^h^
							Individual Predictions																Meta Scores									
Name	pLi ^a^	Class	AD ^b^	DNA Change	Protein Change		1	2	3	4	5	6	7	8	9	10	11	12	13	14	15	16	17	18	19	20	21	22	Score ^e^	Score ^f^	Score ^g^	
***AGO1***	1.00	Very likely dominant	0.999	c.1823C>T	p.Ser608Phe	VUS	**D**	**D**	**D**	**D**	**D**	**H**	**D**	**D**	**PS**	**D**	**D**	**D**	**D**	**D**	**P**	**D**	**D**	**T**	**D**	**0.9**	**D**	**D**		29.20	1.00	
***ARID1A***	1.00	Very likely dominant	1.000	c.6428G>A	p.Arg2143His	VUS	**D**	**D**	**D**	**D**	**D**	**M**	**D**	**D**	**UC**	**D**	**T**	**D**	**D**	**D**	**P**	**D**	**T**	**T**	**D**	**0.91**	**D**	**D**		32.00	1.00	
***ATN1***	1.00	Very likely dominant	0.943	c.2239C>A	p.Pro747Thr	VUS	**D**	**D**	**D**	**D**	**D**	**M**	**D**	**D**	**BP**	**T**	**D**	**D**	**D**	**D**	**P**	**D**	**D**	**D**	**D**	**0.8**	**D**	**D**		26.30	1.00	
***ATP1A1***	1.00	Very likely dominant	0.999	c.1934C>T	p.Ala645Val	VUS	**D**	**D**	**D**	**D**	**D**	**L**	**D**	**D**	**PS**	**D**	**D**	**D**	**D**	**D**	**P**	**D**	**D**	**D**	**D**	**0.95**	**D**	**D**		27.00	1.00	
***CTNNA1***	0.97	Very likely dominant	1.000	c.2429C>T	p.Ser810Phe	VUS	**D**	**D**	**D**	**D**	**N**	**M**	**D**	**D**	**PP**	**D**	**D**	**D**	**D**	**D**	**P**	**D**	**T**	**T**	**D**	**0.83**	**D**	**D**		33.00	1.00	
***EFNB2***	1.00	Very likely dominant	0.998	c.590G>T	p.Ser197Ile	VUS			B	B		**L**		**D**	**PP**	**T**	**T**	**D**	**D**	**T**	**B**	**D**	**T**	**D**	**D**		**D**	**D**		25.40	0.99	
***EXT1***	1.00	Very likely dominant	0.991	c.493C>G	p.Gln165Glu	VUS	**T**	**T**	**P**	B	**D**	**L**	**N**	**D**	**UC**	**T**	**D**	**D**	**D**	**D**	**B**	**D**	**D**	**D**	**D**	**0.81**	**D**	**D**		24.70	0.58	
***FAT1***	0.00	Very likely recessive	0.178	c.2681dupA	p.Ile895AspfsTer2	VUS																										0.54
***FOXA2***	0.97	Likely dominant	0.792	c.779G>C	p.Arg260Pro	VUS		**D**			**D**			**D**	**PS**	**D**		**D**	**D**	**D**	**LP**	**D**	**D**	**D**	**D**	**0.99**	**D**	**D**		35.00	1.00	
***GDF7***	0.00	Very likely dominant	0.991	c.1081C>T	p.Leu361Phe	VUS	**D**	**D**	**D**	**D**	**N**	**M**	**D**	**D**	**UC**	**D**	**D**	**D**	**D**	**D**	**P**	**N**	**D**	**D**	**D**	**0.86**	**D**	**T**		27.00	1.00	
***HAND1***	0.01	Very likely dominant	0.839	c.328A>C	p.Ile110Leu	VUS	**T**	**T**	**D**	**D**	**N**	**N**	**N**	**D**	**UC**	**D**	**D**	**D**	**D**	**D**	**P**	**D**	**D**	**D**	**D**	**0.9**	**D**	**D**		25.90	0.99	
***HIF1A***	1.00	Very likely dominant	1.000	c.806G>A	p.Arg269Gln	VUS	**D**	**D**	**D**	**D**	**D**	**M**	**D**	**D**	**PM**	**D**	**D**	**D**	**D**	**D**	**P**	**D**	**T**	**T**	**D**	**0.85**	**D**	**D**		28.40	1.00	
***HOXB3***	0.00	Very likely dominant	0.988	c.374T>G	p.Leu125Arg	VUS	**T**	**T**	**D**	**D**		**M**	**D**	**D**	**UC**	**T**	**D**	**D**		**D**	**A**		**D**	**D**	**D**	**0.87**	**D**	**D**		26.40	0.99	
***IFT172***	0.00	Very likely recessive	0.152	c.4096G>T	p.Asp1366Tyr	VUS	**D**	**D**	**D**	**D**	**D**	**M**	**D**	**D**	**PP**	**T**	**D**	**D**	**D**	**D**	**A**	**D**	**D**	**D**	**D**	**0.93**	**D**	**D**		26.30	1.00	
***KRT17***	0.00	Very likely recessive	0.152	c.695A>G	p.Gln232Arg	VUS	**D**	**D**	B	B	**D**	**M**	**D**	**D**	**UC**	**T**	**D**	**D**	**T**	**D**	**B**	**D**	**D**	**D**	**D**	**0.85**	**D**	**T**		25.10	1.00	
***LAMA5***	0.00	Likely recessive	0.201	c.3520_3521delAG	p.Arg1174AlafsTer19	VUS																										0.55
***LRP5***	0.94	Very likely dominant	0.946	c.3638-1G>A		VUS																							0.999		0.99	
***MAML1***	1.00	Very likely dominant	0.998	c.193C>T	p.Gln65Ter	LP					**A**									**D**		**N**					**D**	**D**		37.00	1.00	0.50
***MEN1***	1.00	Very likely dominant	1.000	c.1565G>T	p.Arg522Leu	VUS								**D**	**UC**	**D**		**D**		**T**		**D**	**D**	**D**		**0.93**	**D**	**D**		23.40	1.00	
***MYH3***	0.00	Likely recessive	0.400	c.52C>T	p.Arg18Trp	VUS		**D**	**D**	**D**		**H**		**D**	**PS**	**D**	**D**	**D**	**D**	**D**	**P**	**D**	**D**	**D**	**D**		**D**	**D**		32.00	1.00	
***NCOR2***	1.00	Very likely dominant	0.999	c.650C>T	p.Pro217Leu	VUS	**D**	**D**			**N**	**L**	**D**	**D**	**BP**	**T**	**D**	**D**	**D**	**D**	**A**	**D**	**T**	**T**	**D**	**0.74**	**T**	**T**		25.80	0.98	
***NXN***	0.37	Dominant or recessive	0.510	c.503A>C	p.Lys168Thr	VUS	**T**	**T**	**D**	**D**		**L**	**D**	**D**	**PM**	**D**	**T**	**D**	**D**	**D**	**A**	**D**	**T**	**D**	**D**	**0.79**	**D**	**D**		24.00	1.00	
***ROBO1***	0.00	Dominant or recessive	0.539	c.2026C>T	p.Arg676Trp	VUS	**D**	**D**	**D**	**D**	**D**	**M**	**D**	**D**	**PM**	**T**	**T**	**D**	**D**	**D**	**P**	**D**	**T**	**T**	**D**	**0.81**	**D**	**D**		27.60	1.00	
***RPGRIP1L***	0.00	Very likely recessive	0.071	c.413A>G	p.Gln138Arg	VUS			**D**	**D**		**M**		**D**	**BM**	**T**		**D**	**D**	**T**	**B**	**D**	**D**	**D**	**D**	**0.67**	**D**	**D**		23.60	1.00	
***RYR1***	0.00	Very likely dominant	0.987	c.9136C>G	p.Leu3046Val	VUS	**D**		**P**	B	**N**	**M**	**N**	**D**	**PP**	**T**	**D**	**D**	**D**	**D**	**A**	**D**	**D**	**T**	**D**	**0.74**	**T**	**T**		22.20	0.99	
***RYR1***	0.00	Very likely dominant	0.987	c.9198C>G	p.Asn3066Lys	VUS	**T**		B	B	**D**	**L**	**D**	**D**	**UC**	**T**	**D**	**D**	**D**	**D**	**P**	**D**	**D**	**D**	**D**	**0.72**	**T**	**T**		20.10	1.00	
***SLC32A1***	1.00	Likely dominant	0.737	c.466G>T	p.Ala156Ser	VUS	**D**	**D**	**D**	**D**	**D**	**M**	**D**	**T**	**PP**	**D**	**T**	**D**	**D**	**T**	**A**	**D**	**T**	**T**	**D**	**0.53**	**T**	**T**		29.70	1.00	
***TENM4***	1.00	Very likely dominant	0.878	c.7916G>A	p.Arg2639Gln	VUS	**D**	**D**	**D**	**D**	**N**	**M**	**N**	**D**	**UC**	**T**	**T**	**D**	**D**	**T**	**B**	**D**	**D**	**D**	**D**	**0.79**	**T**	**T**		28.60	1.00	
***TP53BP2***	0.00	Dominant or recessive	0.438	c.1757C>A	p.Ala586Asp	VUS	**D**	**D**			**N**		**D**	**D**	**UC**	**T**		**D**	**D**	**T**	**LP**	**D**	**T**	**T**	**D**	**0.52**	**D**	**D**		24.00	1.00	
***VWA8***	0.00	Very likely recessive	0.092	c.2003G>A	p.Arg668Gln	VUS (leaning LP)	**D**	**D**	**D**	**D**	**A**	**M**	**D**	**D**	**UC**	**T**	**T**	**T**	**D**	**D**	**A**	**D**	**T**	**T**	**T**	**0.73**	**T**	**D**		27.90	1.00	
***ZNF319***	0.51	Likely dominant	0.744	c.190C>T	p.Gln64Ter	LP														**D**		**N**					**D**	**D**		38.00	0.99	0.45

^a^ pLI—probability of being loss-of-function (LoF) intolerant; pLI ≥ 0.9 indicates LoF intolerance (gnomAD v4.1.0, https://gnomad.broadinstitute.org/, accessed on 1 June 2025). ^b^ Probability of being autosomal dominant (AD)—probability of a gene being associated with autosomal dominant conditions (DOMINO, https://domino.iob.ch/index.php, accessed on 1 June 2025). ^c^ ACMG, the American College of Medical Genetics and Genomics; LP, likely pathogenic, VUS, variant of uncertain significance. ^d^ Pathogenicity of missense variants predicted using 22 in silico tools: (1) SIFT, (2) SIFT4G, (3) PolyPhen-2_HDIV, (4) PolyPhen-2_HVAR, (5) MutationTaster, (6) MutationAssessor, (7) PROVEAN, (8) M-CAP, (9) MutPred2, (10) PrimateAI, (11) DEOGEN2, (12) ClinPred, (13) LIST-S2, (14) ESM1b, (15) AlphaMissense, (16) fathmm-XF, (17) MetaSVM, (18) MetaLR, (19) MetaRNN, (20) REVEL, (21) BayesDel_addAF, and (22) BayesDel_noAF (https://www.dbnsfp.org/home, accessed on 1 June 2025). Abbreviations: A, ambiguous; B, benign; BM, benign moderate; BP, benign supporting; D, damaging/deleterious; H, high; L, low; LP, likely pathogenic; M, medium; N, neutral; P, pathogenic; PM, pathogenic moderate; PP, pathogenic supporting; PS, pathogenic strong; T, tolerated; UC, uncertain. Color coding: red—pathogenic/deleterious predictions; green—benign/neutral/tolerated/uncertain predictions. REVEL (20): score range 0–1; ≥0.644 = pathogenic supporting; 0.643 − 0.291 = neutral; ≤0.290 = benign supporting. ^e^ ADA—adaptive boosting score predicting splice-affecting variants; values close to 1 indicate high probability (dbNSFP v5.2). ^f^ CADD—Combined Annotation Dependent Depletion; only missense variants with CADD ≥ 20 were included. Variants with scores above 20 rank among the top 1% most deleterious possible substitutions in the human genome (dbNSFP v5.2). ^g^ DANN—Deleterious Annotation of genetic variants using Neural Networks; scores > 0.96 indicate high pathogenicity (dbNSFP v5.2). ^h^ MutPred-LOF—applied for frameshift and stop-gain variants (http://mutpred2.mutdb.org/mutpredlof/, accessed on 1 June 2025); scores ≥ 0.50 suggest pathogenicity with a false positive rate of 10%.

**Table 3:** Associations of common variants within loci for genes carrying rare likely pathogenic variants identified in ns-CL/P patients.

	Number of SNVs Plotted	The Most Significant SNV Within a Locus					
Gene ^a^		rs Number	Location ^b^	Position Relative to Gene ^c^	MAF ^d^	*p*_trend_ Value ^e^	Allelic OR ^f^
***AGO1***	11	rs6682769	chr1: 35860595	22.6 kb upstream, intergenic	0.06	3.36 × 10^−2^	1.57
***ARID1A***	20	rs4466675	chr1: 26693521	2.5 kb upstream, intergenic	0.48	2.05 × 10^−2^	0.79
***ATN1***	43	rs10744724	chr12: 6956118	13.8 kb downstream, within *PTPN6*	0.06	6.70 × 10^−3^	0.50
***ATP1A1***	29	rs766429	chr1: 116495418	90.6 kb downstream, intergenic	0.21	1.76 × 10^−1^	0.84
***CTNNA1***	33	rs12108892	chr5: 138963941	28.9 kb downstream, within *SIL1*	0.39	8.13 × 10^−2^	0.83
***EFNB2***	91	rs7490929	chr13: 106431374	58.4 kb downstream, intergenic	0.07	3.75 × 10^−2^	1.51
***EXT1***	**128**	**rs7837891**	**chr8: 117807339**	**exonic (synonymous variant)**	**0.44**	**9.56 × 10^−4^**	**0.70**
***FAT1***	93	rs28647489	chr4: 186609868	exonic (missense variant)	0.14	3.18 × 10^−2^	0.71
***FOXA2***	35	rs2404167	chr20: 22528795	52.2 kb downstream, intergenic	0.10	3.77 × 10^−2^	1.62
***GDF7***	64	rs340596	chr2: 20689994	10.8 kb downstream, within *C2orf43*	0.43	7.43 × 10^−3^	0.76
***HAND1***	86	rs283438	chr5: 154494000	15.8 kb upstream, intergenic	0.17	3.06 × 10^−2^	1.34
***HIF1A***	35	rs17099248	chr14: 61832287	84.0 kb downstream, intergenic	0.12	2.57 × 10^−2^	0.68
***HOXB3***	52	rs890435	chr17: 48642037	51.8 kb upstream, intergenic	0.40	1.03 × 10^−2^	1.31
***IFT172***	33	rs3811644	chr2: 27579938	90.2 kb upstream, within *SPATA31H1*	0.21	2.49 × 10^−1^	0.86
***KRT17***	31	rs7503702	chr17: 41609234	10.2 kb upstream, intergenic	0.40	1.04 × 10^−2^	1.30
***LAMA5***	72	rs4925238	chr20: 62431508	64.2 kb upstream, intergenic	0.06	1.79 × 10^−2^	1.61
***LRP5***	51	rs314779	chr11: 68330358	intronic	0.23	2.76 × 10^−2^	0.76
***MAML1***	**41**	**rs28564876**	**chr5: 179651067**	**81.8 kb upstream, intergenic**	**0.41**	**9.34 × 10^−4^**	**1.43**
***MEN1***	29	rs10897529	chr11: 64829444	18.9 kb upstream, within *CDC42BPG*	0.35	2.31 × 10^−1^	1.14
***MYH3***	30	rs6503319	chr17: 10670842	13.5 kb upstream, intergenic	0.25	3.90 × 10^−2^	1.27
***NCOR2***	154	rs7132377	chr12: 124667448	99.8 kb upstream, intergenic	0.09	4.63 × 10^−3^	0.56
***NXN***	**127**	**rs8081951**	**chr17: 933301**	**intronic**	**0.18**	**2.52 × 10^−4^**	**1.62**
***ROBO1***	176	rs1865862	chr3: 78754700	intronic	0.28	1.25 × 10^−2^	0.74
***RPGRIP1L***	58	rs1421085	chr16: 53767042	63.2 kb upstream, within *FTO*	0.48	2.49 × 10^−3^	1.36
***RYR1***	59	rs4802351	chr19: 38358156	72.5 kb upstream, within *CATSPERG*	0.14	2.50 × 10^−2^	0.70
***SLC32A1***	41	rs2902891	chr20: 38681144	43.3 kb upstream, intergenic	0.30	7.65 × 10^−2^	1.22
***TENM4***	312	rs12362098	chr11: 78857065	intronic	0.16	2.07 × 10^−3^	1.50
***TP53BP2***	**51**	**rs7535882**	**chr1: 223857777**	**11.8 kb upstream, intergenic**	**0.07**	**1.16 × 10^−3^**	**1.80**
***VWA8***	118	rs12585194	chr13: 41977432	16.3 kb upstream, within *VWA8−AS1*	0.13	1.43 × 10^−2^	1.46
***ZNF319***	48	rs17241022	chr16: 57920621	74.1 kb downstream, within *CNGB1*	0.10	4.40 × 10^−2^	0.68

Data from a genome-wide association study (GWAS) on ns-CL/P in the Polish population, as previously reported [39]. Number of tested genes: 30; number of tested Single Nucleotide Variants (SNVs): 2151. ^a^ Gene ± 100 kb. ^b^ GRCh38/hg38 genome assembly. ^c^ According to MANE Select Plus Clinical: representative transcript from RefSeq & GENCODE. ^d^ MAF, minor allele frequency based on sample-wide allele frequencies. ^e^ *p*_trend_ < 1.67 × 10^−3^ (0.05/30 genes) considered statistically significant; none remained significant after correction by the number of tested SNVs (*p*_trend_ < 2.32 × 10^−5^). ^f^ OR, odds ratio.

**Table 4:** Gene Ontology (GO) Biological Process enrichment analysis of ns-CL/P associated genes.

Biological Process	GO Term	*p* Value	*p*_adj_ Value ^a^	Genes
tissue morphogenesis	GO:0048729	1.34 × 10^−10^	1.21 × 10^−6^	*EFNB2; EXT1; FAT1; GDF7; HAND1; HIF1A; IFT172; KRT17; LAMA5; LRP5; ROBO1; RPGRIP1L*
embryonic morphogenesis	GO:0048598	1.45 × 10^−10^	1.31 × 10^−6^	*EXT1; FOXA2; GDF7; HAND1; HIF1A; HOXB3; IFT172; LAMA5; LRP5; MYH3; RPGRIP1L; TENM4*
animal organ morphogenesis	GO:0009887	2.48 × 10^−10^	2.24 × 10^−6^	*CTNNA1; EFNB2; EXT1; FAT1; GDF7; HAND1; HIF1A; HOXB3; IFT172; LAMA5; LRP5; ROBO1; RPGRIP1L; RYR1*
morphogenesis of an epithelium	GO:0002009	3.04 × 10^−10^	2.75 × 10^−6^	*EFNB2; EXT1; FAT1; GDF7; HAND1; HIF1A; IFT172; KRT17; LAMA5; LRP5; RPGRIP1L*
circulatory system development	GO:0072359	9.43 × 10^−10^	8.52 × 10^−6^	*AGO1; EFNB2; EXT1; HAND1; HIF1A; HOXB3; IFT172; LRP5; MAML1; NXN; ROBO1; RPGRIP1L; RYR1; TENM4*
regionalization	GO:0003002	1.29 × 10^−9^	1.17 × 10^−5^	*EXT1; FOXA2; HAND1; HIF1A; HOXB3; IFT172; LAMA5; LRP5; ROBO1; RPGRIP1L*
tissue development	GO:0009888	1.98 × 10^−9^	1.79 × 10^−5^	*EFNB2; EXT1; FAT1; FOXA2; GDF7; HAND1; HIF1A; HOXB3; IFT172; KRT17; LAMA5; LRP5; MAML1; ROBO1; RPGRIP1L; RYR1; TENM4*
pattern specification process	GO:0007389	3.33 × 10^−9^	3.00 × 10^−5^	*EXT1; FOXA2; HAND1; HIF1A; HOXB3; IFT172; LAMA5; LRP5; ROBO1; RPGRIP1L*
embryo development	GO:0009790	1.14 × 10^−8^	1.03 × 10^−4^	*EXT1; FOXA2; GDF7; HAND1; HIF1A; HOXB3; IFT172; LAMA5; LRP5; MYH3; NXN; RPGRIP1L; TENM4*
heart development	GO:0007507	2.95 × 10^−8^	2.67 × 10^−4^	*EFNB2; EXT1; HAND1; HIF1A; IFT172; MAML1; ROBO1; RPGRIP1L; RYR1; TENM4*
gland development	GO:0048732	4.08 × 10^−8^	3.69 × 10^−4^	*EXT1; GDF7; HIF1A; HOXB3; LAMA5; LRP5; NCOR2; ROBO1; RPGRIP1L*
central nervous system development	GO:0007417	4.52 × 10^−8^	4.08 × 10^−4^	*ATN1; EXT1; GDF7; HIF1A; HOXB3; IFT172; LRP5; NCOR2; ROBO1; RPGRIP1L; SLC32A1; TENM4*
epithelial tube morphogenesis	GO:0060562	6.40 × 10^−8^	5.78 × 10^−4^	*EFNB2; EXT1; GDF7; HAND1; HIF1A; IFT172; LAMA5; LRP5*
tube development	GO:0035295	8.68 × 10^−8^	7.84 × 10^−4^	*AGO1; EFNB2; EXT1; GDF7; HAND1; HIF1A; HOXB3; IFT172; LAMA5; LRP5; ROBO1; RPGRIP1L*
tube morphogenesis	GO:0035239	9.33 × 10^−8^	8.43 × 10^−4^	*AGO1; EFNB2; EXT1; GDF7; HAND1; HIF1A; HOXB3; IFT172; LAMA5; LRP5; ROBO1*
anatomical structure formation involved in morphogenesis	GO:0048646	2.19 × 10^−7^	1.97 × 10−^3^	*AGO1; EFNB2; EXT1; FOXA2; GDF7; HAND1; HIF1A; HOXB3; IFT172; MYH3; ROBO1; TENM4*
epithelium development	GO:0060429	3.06 × 10^−7^	2.77 × 10^−3^	*EFNB2; EXT1; FAT1; GDF7; HAND1; HIF1A; IFT172; KRT17; LAMA5; LRP5; ROBO1; RPGRIP1L*
head development	GO:0060322	3.38 × 10^−7^	3.05 × 10^−3^	*EXT1; GDF7; HIF1A; HOXB3; IFT172; MYH3; NCOR2; ROBO1; RPGRIP1L; SLC32A1*
brain development	GO:0007420	2.10 × 10^−6^	1.90 × 10^−2^	*EXT1; GDF7; HIF1A; HOXB3; IFT172; NCOR2; ROBO1; RPGRIP1L; SLC32A1*
skeletal system development	GO:0001501	2.20 × 10^−6^	1.99 × 10^−2^	*EXT1; HAND1; HIF1A; HOXB3; IFT172; LAMA5; LRP5; RYR1*
camera-type eye morphogenesis	GO:0048593	2.73 × 10^−6^	2.47 × 10^−2^	*FAT1; HIF1A; IFT172; LRP5; RPGRIP1L*
regulation of cell differentiation	GO:0045595	3.88 × 10^−6^	3.50 × 10^−2^	*ARID1A; CTNNA1; EFNB2; FOXA2; GDF7; HIF1A; HOXB3; LRP5; MAML1; MEN1; ROBO1; TENM4*
heart morphogenesis	GO:0003007	4.78 × 10^−6^	4.32 × 10^−2^	*EXT1; HAND1; HIF1A; IFT172; ROBO1; RYR1*
morphogenesis of embryonic epithelium	GO:0016331	5.31 × 10^−6^	4.80 × 10^−2^	*GDF7; HAND1; HIF1A; IFT172; LAMA5*

^a^ Multiple testing procedure: Bonfferoni correction. All significantly enriched GO biological process terms are presented (*p*_adj_ < 0.05). Analysis performed using WEB-based GEne SeT AnaLysis Toolkit (WebGestalt 2024; https://www.webgestalt.org/ (accessed on 1 June 2025)).

**Table 5:** Clinical characteristics of patients with ns-CL/P.

	All Patients (n = 58) ^a^	
	N	%
**GENDER DISTRIBUTION**		
Males	36	62.07
Females	22	37.93
**CLEFT TYPE**		
**Cleft lip with or without cleft palate**	**48**	**82.76**
Unilateral—right side	10	17.24
Unilateral—left side	21	36.21
Bilateral	15	25.86
Side unknown	2	3.45
**Cleft lip only**	**10**	**17.24**
Unilateral—right side	3	5.17
Unilateral—left side	2	3.45
Bilateral	1	1.72
Side unknown	4	6.90
**ASSOCIATED ANOMALIES**	**16**	**27.59**
Kidney defects ^b^	2	3.45
**POSITIVE FAMILY HISTORY**	**19**	**32.76**
Immediate family (parents, siblings)	6	10.34
Extended family	13	22.41

^a^ One sibling pair included. ^b^ Bifid left renal pelvis or horseshoe kidney.

## Data Availability

The de-identified datasets generated through this study can be provided by the corresponding author upon request.

## Supplementary Materials

The following supporting information can be downloaded at: https://www.mdpi.com/article/10.3390/ijms262412111/s1.

## Abbreviations

The following abbreviations are used in this manuscript:

AD	Autosomal Dominant
ADA	Adaptive Boosting
CADD	Combined Annotation Dependent Depletion
DANN	Deleterious Annotation of Genetic Variants Using Neural Networks
DNV	De Novo variant
GO	Gene Ontology
GWAS	Genome-Wide Association Study
LD	Linkage Disequilibrium
LoF	Loss of Function
MAF	Minor Allele Frequency
NFE	European non-Finnish
NGS	Next-Generation Sequencing
ns-CL	Non-Syndromic Cleft Lip
ns-CL/P	Non-Syndromic Cleft Lip with or without Cleft Palate
ns-CLP	Non-Syndromic Cleft Lip with Cleft Palate
ORA	Overrepresentation Enrichment Analysis
P/LP	Pathogenic or Likely Pathogenic
pLI	Probability of Being Loss-of-Function Intolerant
SNV	Single Nucleotide Variants
VUS	Variant of Uncertain Significance
WES	Whole-Exome Sequencing
WGS	Whole-Genome Sequencing
