# Supplementary material for: Expanding the Genetic Spectrum of Non-Syndromic Cleft Lip and Palate Through Whole-Exome Sequencing
Source: Int J Mol Sci. 2025 Dec 16;26(24):12111. doi: 10.3390/ijms262412111 (PMC12733283; doi:10.3390/ijms262412111)
Supplement: Supplementary file 1 [file ijms-26-12111-s001.zip › Supplementary Figure Legends.pdf]

Supplementary Figure Legends

Supplementary Figure S1. Regional plot for the *EXT1* locus (gene ±100 kb).

Data were retrieved from a genome-wide association study (GWAS) of ns-CL/P conducted in the Polish population. Details of the study design, analysis, and results were previously described [70,71]. The left Y-axis shows Cochran–Armitage trend test p-values ($-\log_{10}$ scale) of individual single nucleotide variants (SNVs) plotted against their chromosomal position (Mb) on the X-axis. The right Y-axis shows the local recombination rate estimated from the HapMap CEU population. The top SNV in the region, rs7837891, is highlighted in purple. All other SNVs are colour-coded according to the strength of their pairwise linkage disequilibrium (LD, r^2) with the top SNV. At the bottom, genes located in the region are shown with their exon–intron structure, transcriptional direction, and genomic coordinates (GRCh37/hg19). Regional plots were generated using LocusZoom v1.1.

Supplementary Figure S2. Regional plot for the *MAML1* locus (gene ±100 kb).

Data were retrieved from a genome-wide association study (GWAS) of ns-CL/P conducted in the Polish population. Details of the study design, analysis, and results were previously described [70,71]. The left Y-axis shows Cochran–Armitage trend test p-values ($-\log_{10}$ scale) of individual single nucleotide variants (SNVs) plotted against their chromosomal position (in Mb) on the X-axis. The right Y-axis shows the local recombination rate estimated from the HapMap CEU population. The top SNV in the region, rs28564876, is highlighted in purple. All other SNVs are colour-coded according to the strength of their pairwise linkage disequilibrium (LD, r^2) with the top SNV. At the bottom, genes located in the region are shown with their exon–intron structure, transcriptional direction, and genomic coordinates (GRCh37/hg19). Regional plots were generated using LocusZoom v1.1.

Supplementary Figure S3. Regional association plot for the *NXN* locus (±100 kb).

Data were retrieved from a genome-wide association study (GWAS) of ns-CL/P conducted in the Polish population. Details of the study design analysis, and results were previously described [70,71]. The left Y-axis shows Cochran–Armitage trend test p-values ($-\log_{10}$ scale) of individual single nucleotide variants (SNVs) plotted against their chromosomal position (in Mb) on the X-axis. The right Y-axis shows the local recombination rate estimated from the HapMap CEU population. The top SNV in the region, rs8081951, is highlighted in purple. All other SNVs are colour-coded according to the strength of their pairwise linkage disequilibrium (LD, r^2) with the top SNV. At the bottom,

genes located in the region are shown with their exon–intron structure, transcriptional direction, and genomic coordinates (GRCh37/hg19). Regional plots were generated using LocusZoom v1.1.

Supplementary Figure S4. Regional plot for the *TP53BP2* locus (gene ±100 kb).

Data were retrieved from a genome-wide association study (GWAS) of ns-CL/P conducted in the Polish population. Details of the study design, analysis, and results were previously described [70,71]. The left Y-axis shows Cochran–Armitage trend test p-values ($-\log_{10}$ scale) of individual single nucleotide variants (SNVs) plotted against their chromosomal position (in Mb) on the X-axis. The right Y-axis shows the local recombination rate estimated from the HapMap CEU population. The top SNV in the region, rs7535882, is highlighted in purple. All other SNVs are colour-coded according to the strength of their pairwise linkage disequilibrium (LD, r^2) with the top SNV. At the bottom, genes located in the region are shown with their exon–intron structure, transcriptional direction, and genomic coordinates (GRCh37/hg19). Regional plots were generated using LocusZoom v1.1.

Supplementary Figure S5. Gene Ontology (GO) Slim summary of genes harbouring rare likely pathogenic variants detected by whole-exome sequencing (WES) in ns-CL/P patients.

Bar plots show the number of genes from the input list overlapping with annotated genes in GO Slim terms for (A) biological process and (B) molecular function categories. The analysis was performed using the WebGestalt tool (WEB-based Gene SeT AnaLysis Toolkit, http://www.webgestalt.org/).

Supplementary Figure S6. Directed acyclic graph (DAG) of significantly enriched biological process Gene Ontology (GO) terms for genes harbouring rare likely pathogenic variants detected by whole-exome sequencing (WES) in ns-CL/P patients.

Nodes represent enriched GO terms within the biological process category, with box colour intensity corresponding to the significance of enrichment (adjusted p-value). Edges indicate hierarchical relationships between GO terms. The top five most significantly over-represented terms are "tissue morphogenesis," "embryonic morphogenesis," "animal organ morphogenesis," "morphogenesis of an epithelium," and "circulatory system development" ($p_{adj} \leq 8.52E\text{-}06$). The analysis was performed using the WebGestalt tool (WEB-based Gene SeT AnaLysis Toolkit, http://www.webgestalt.org/).
