# Supplementary figures and images for: Expanding the Genetic Spectrum of Non-Syndromic Cleft Lip and Palate Through Whole-Exome Sequencing
Source: Int J Mol Sci. 2025 Dec 16;26(24):12111. doi: 10.3390/ijms262412111 (PMC12733283; doi:10.3390/ijms262412111)

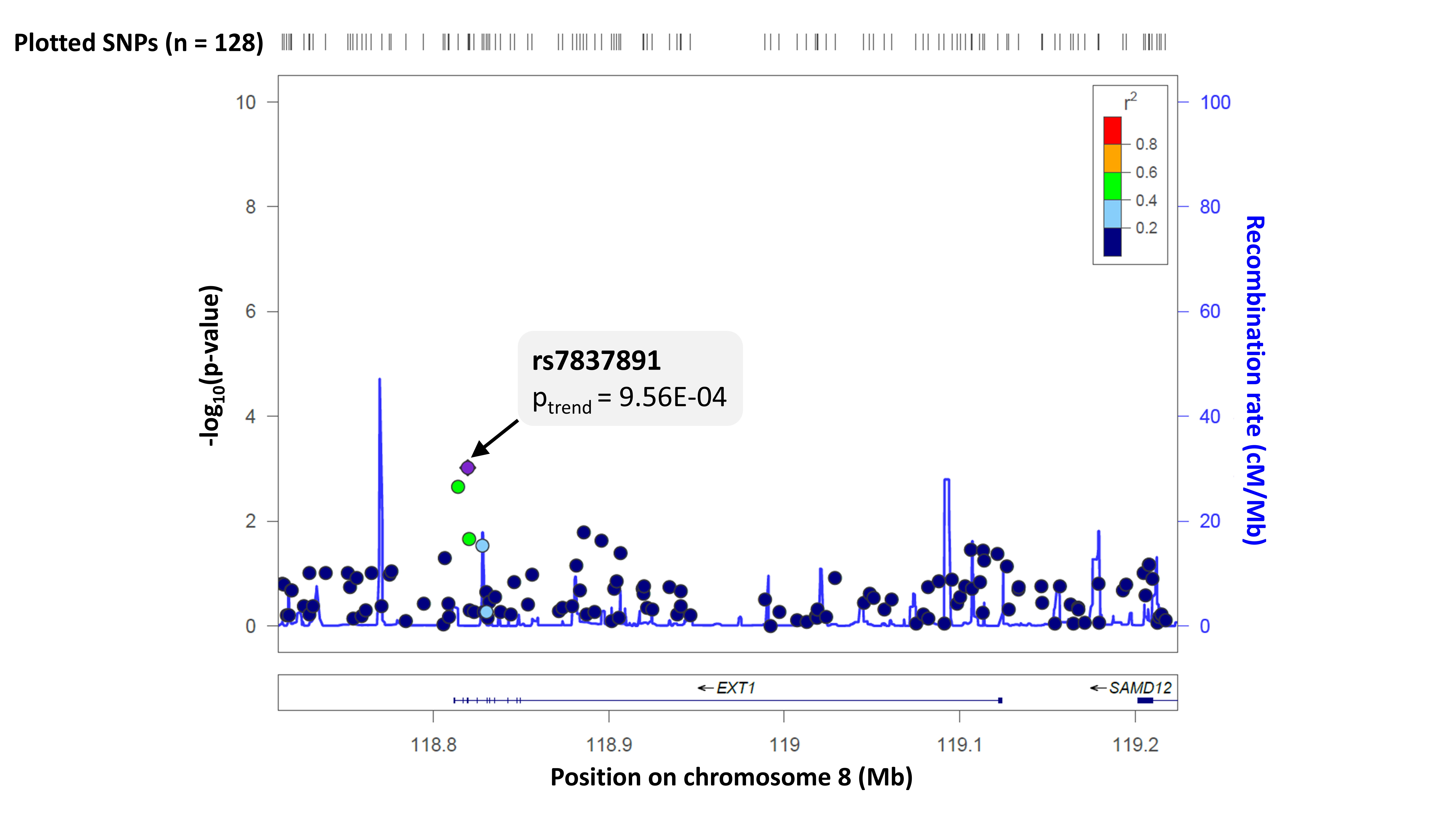

Supplement: Supplementary file 1 [file ijms-26-12111-s001.zip › Figure S1.jpg]

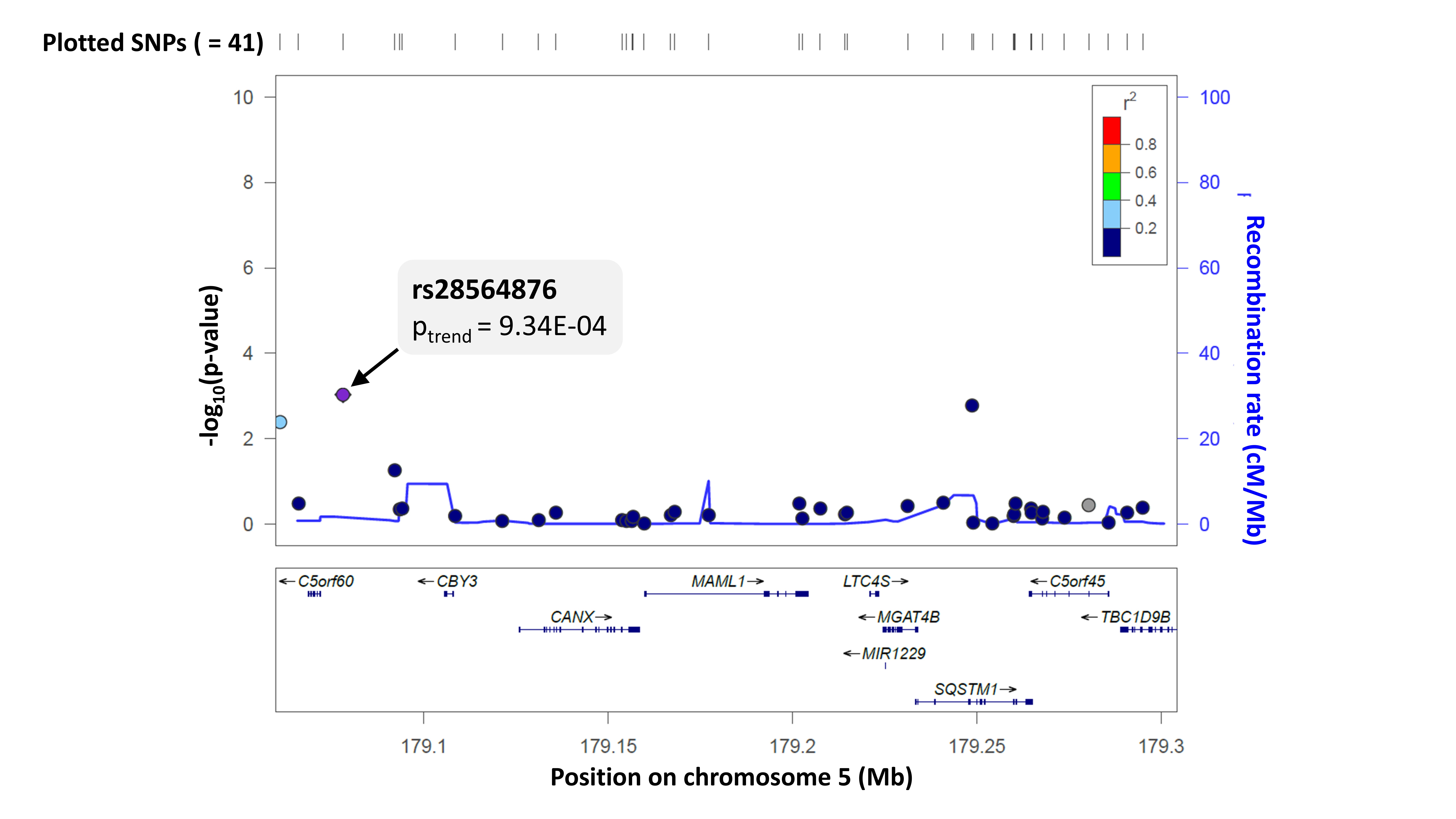

Supplement: Supplementary file 1 [file ijms-26-12111-s001.zip › Figure S2.jpg]

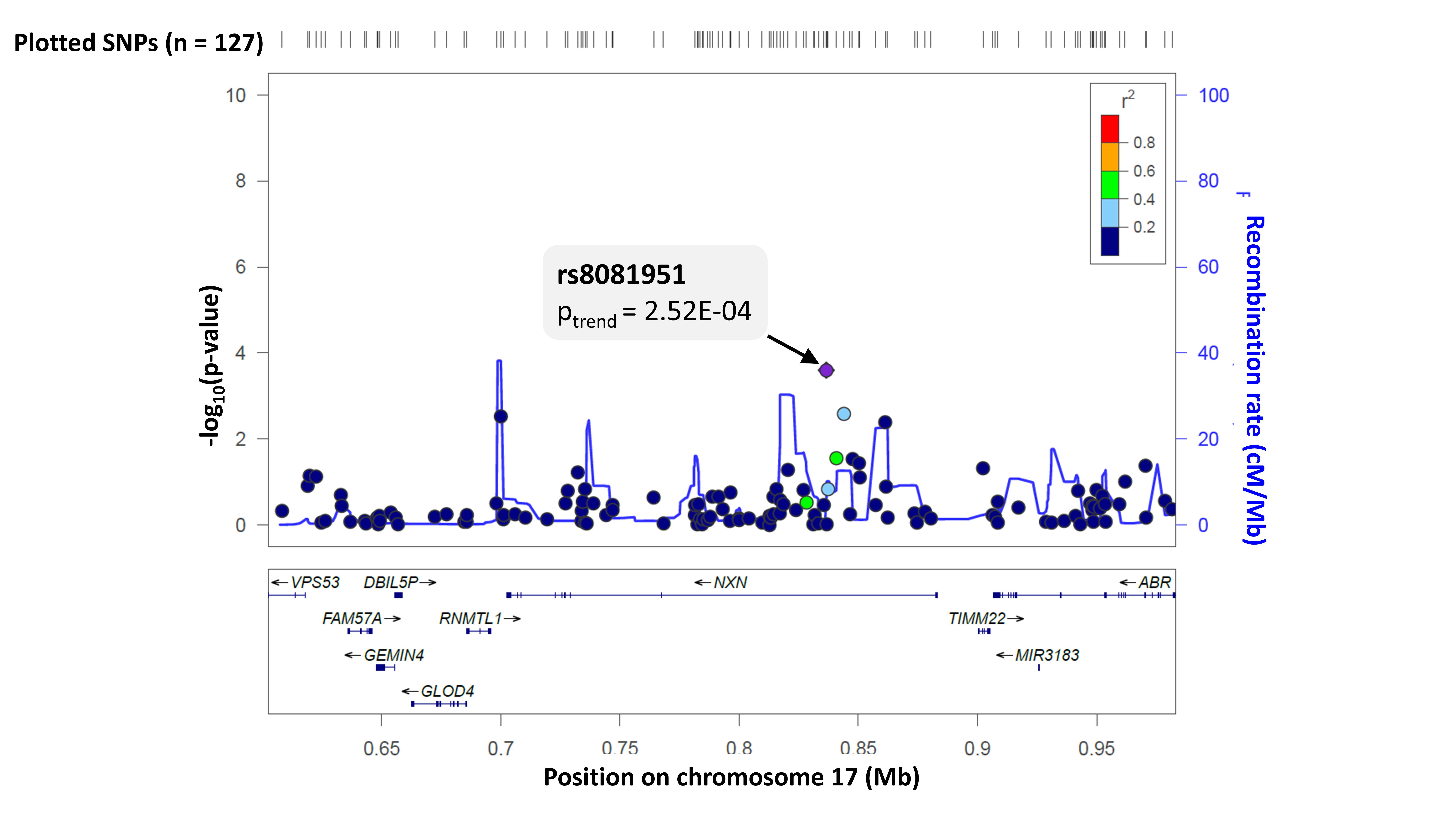

Supplement: Supplementary file 1 [file ijms-26-12111-s001.zip › Figure S3.jpg]

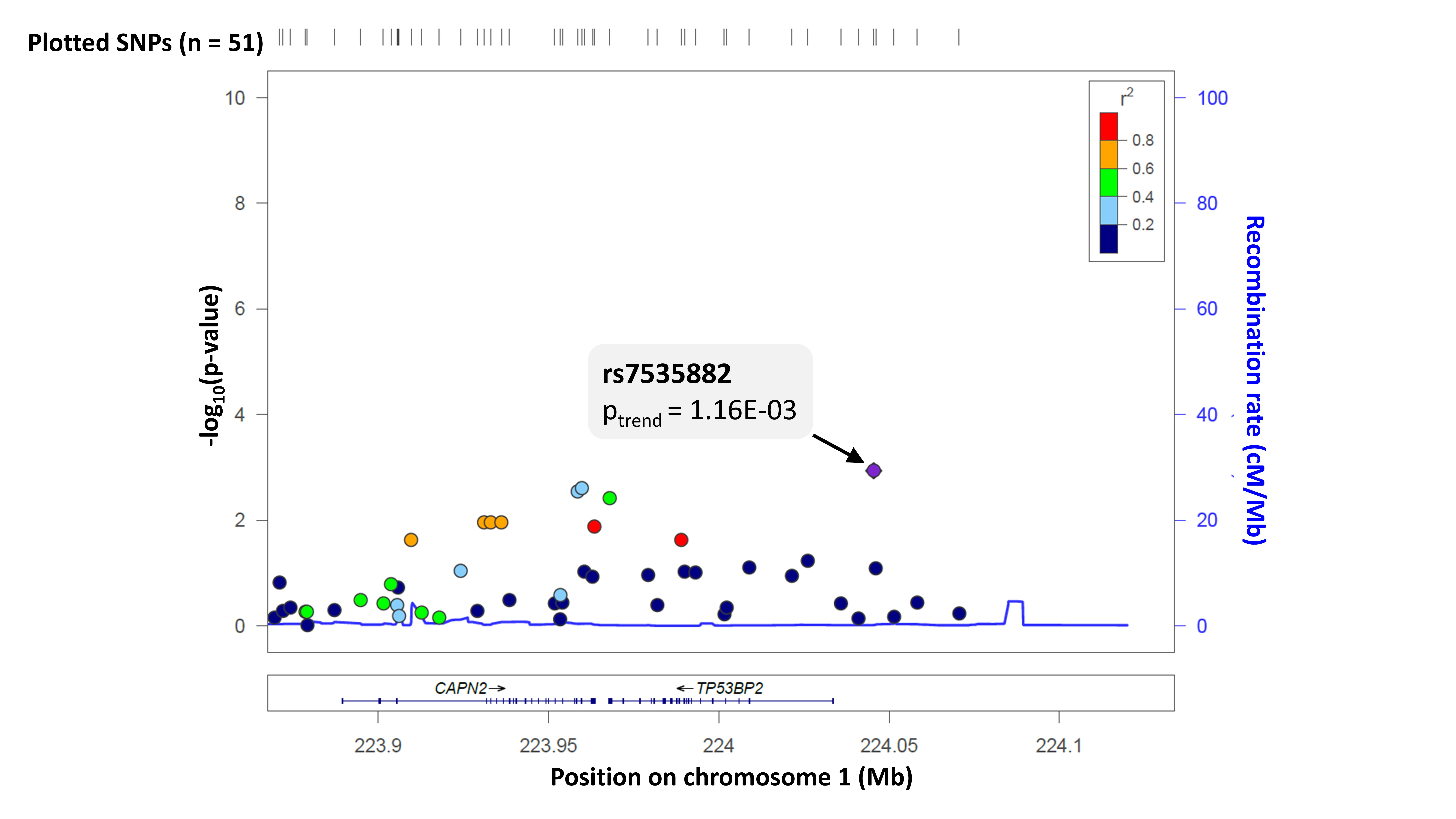

Supplement: Supplementary file 1 [file ijms-26-12111-s001.zip › Figure S4.jpg]

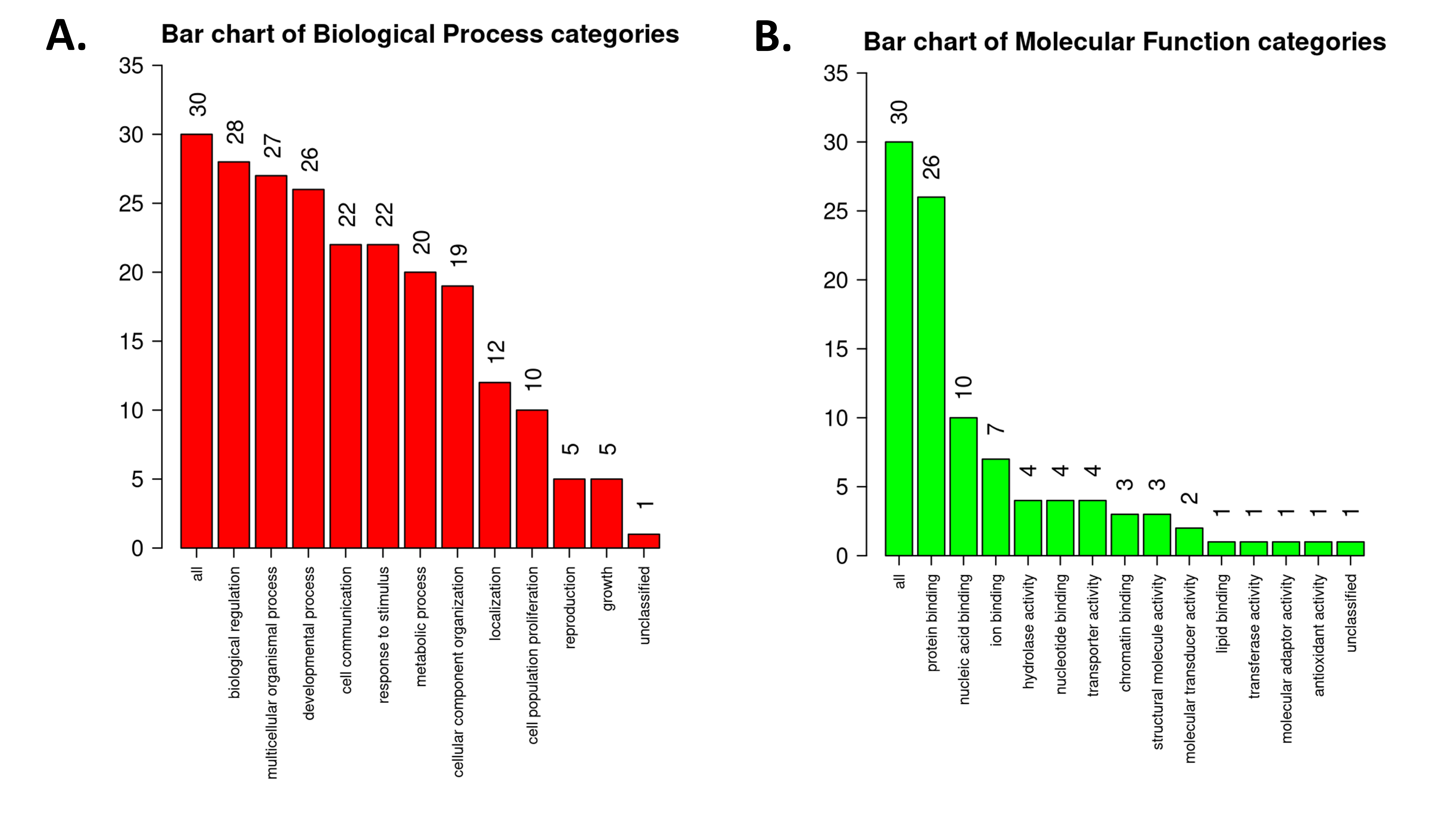

Supplement: Supplementary file 1 [file ijms-26-12111-s001.zip › Figure S5.jpg]

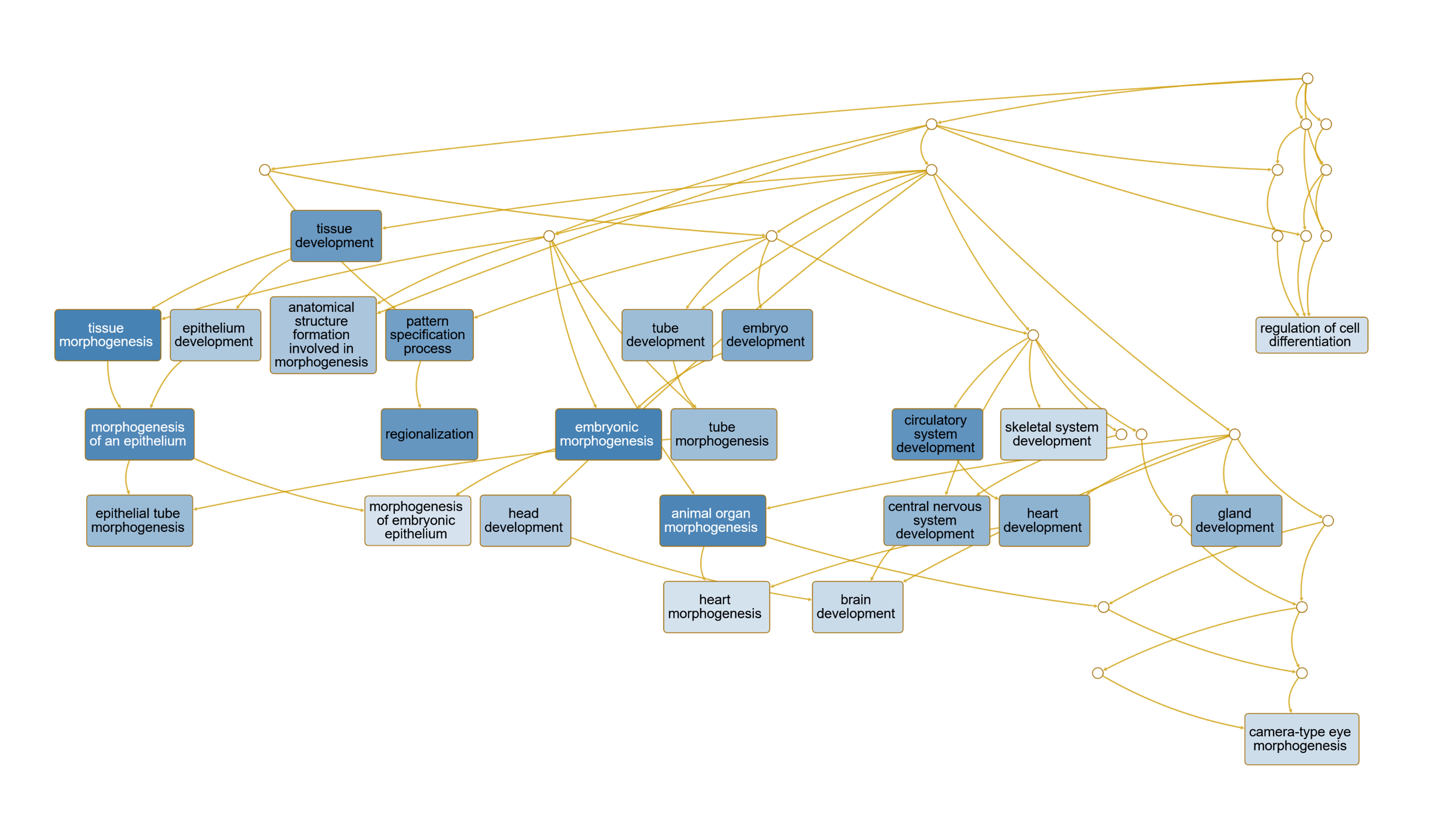

Supplement: Supplementary file 1 [file ijms-26-12111-s001.zip › Figure S6.jpg]
